# Noise Analysis and Suppression Methods for the Front-End Readout Circuit of a Microelectromechanical Systems Gyroscope

**DOI:** 10.3390/s24196283

**Published:** 2024-09-28

**Authors:** Chunhua He, Yingyu Xu, Xiaoman Wang, Heng Wu, Lianglun Cheng, Guizhen Yan, Qinwen Huang

**Affiliations:** 1 School of Computer, Guangdong University of Technology, Guangzhou 510006, China; hechunhua@pku.edu.cn (C.H.); 2112205284@mail2.gdut.edu.cn (Y.X.); 2112205067@mail2.gdut.edu.cn (X.W.); llcheng@gdut.edu.cn (L.C.); 2 Science and Technology on Reliability Physics and Application Technology of Electronic Component Laboratory, China Electronic Product Reliability and Environmental Testing Research Institute, Guangzhou 511370, China; 3 National Key Laboratory of Science and Technology on Micro/Nano Fabrication, Institute of Microelectronics, Peking University, Beijing 100871, China; gzyan@pku.edu.cn

**Keywords:** MEMS gyroscope, noise analysis, detection circuit, control circuit, noise suppression

## Abstract

Circuit noise is a critical factor that affects the performances of an MEMS gyroscope. Therefore, it is essential to analyze and suppress the noises in the key analog circuits, which are the main noise sources. This study presents an optimized front-end readout circuit and noise suppression methods. First, the noise analysis of the front-end readout circuit is carried out with theoretical derivation to clarify the main noise contributors. To suppress the output noise, an improved readout circuit based on the T-resistor networks is proposed, and the corresponding noise equation is derived in detail. In addition, the noise analysis of the critical circuits of the detection and control system, such as the inverting amplifiers, the first-order low-pass filters, and the first-order high-pass filters, is carried out, and the noise suppression strategy with the optimization of the resistances and is proposed. Taking the inverting amplifier as an example, the theoretical derivation is verified by measuring and comparing the output noises of different resistance schemes. In addition, the output noises of the gyroscope before and after circuit optimization are measured. Experimental results demonstrate that the output noise with the circuit optimization is reduced from 60 μV/Hz^1/2^ to 30 μV/Hz^1/2^ and the bias instability is reduced from 3.8 deg/h to 1.38 deg/h. In addition, the ARW is significantly improved from 0.035 deg/h^1/2^ to 0.018 deg/h^1/2^, which indicates that the proposed noise analysis and suppression methods are effective and feasible.

## 1. Introduction

A microelectromechanical systems (MEMS) gyroscope is a crucial component of an inertial measurement unit (IMU) [1,2]. Nowadays, industrial robots, drones, unmanned vehicles, and other intelligent terminals with IMUs have been widely developed and applied in industrial production and social life. For unmanned terminals, localization and attitude sensing provide basic motion information for their operation and control [3,4]. In particular, the MEMS gyroscope provides the angular velocity for attitude recognition and combined navigation [5]. When a gyroscope vibrating along the *x*-axis rotates around the *z*-axis with a certain angular velocity, a Coriolis force will be generated, causing the mass block to vibrate along the *y*-axis [6,7]. The distance between the detection electrode and the mass block varies proportionally to the angular velocity, causing a change in the capacitance [8]. Therefore, the angular velocity can be calculated by detecting the small capacitance change with a front-end readout circuit [9,10].

In IMUs, circuit noise is an important factor that affects and limits gyroscopes’ performances [11,12,13]. Noise mainly includes 1/f noise, shot noise, Johnson noise, mechanical thermal noise, etc. [14,15]. Noise is a random signal that interferes with the useful signals of the detection and control system. When the signal-to-noise ratio (SNR) is low, the useful signals may be significantly disrupted or even overwhelmed by the noise [16]. Since voltage and current noise are stochastic, it is essential to select a suitable statistical method for noise analysis and evaluate their impacts on the gyroscope’s performances. In general, the distribution of random signal energy can be characterized through the power spectral density (PSD) [17]. Additionally, nonsmooth noise can be differential processed to achieve smoothing [18]. The magnitude of the noise can be estimated with the mean squared error (MSE), and Allan variance can be utilized to effectively separate noise sources, particularly for nonstationary stochastic processes [19,20].

After identifying the main noise contributors, it is necessary to adopt appropriate measures to suppress the noises. Currently, noise reduction techniques can be classified as either preprocessing techniques or post-processing techniques. Preprocessing techniques are mainly realized by optimizing the circuit structure, resistance, capacitance, and chip selection, which can suppress the noise from the source [21,22,23]. For example, Mengxiang Liu et al. designed a two-stage charge amplifier instead of a transimpedance amplifier, which greatly reduces the input current noise and the overall phase shift of the loop introduced by the transimpedance [24]. Feng Bu et al. developed a noise model for force-to-rebalance closed-loop detection by analyzing the transfer path of electro-feedback noise in the electromechanical amplitude modulation detection circuit of an MEMS gyroscope. After noise optimization, the angle random walk (ARW) was reduced from 0.148°/√h to 0.017°/√h, improving performance by about 8.7 times [25]. Guoming Xia et al. implemented a high-precision frequency readout method and CMOS integrated circuit for a vibrating beam accelerometer using the sigma-delta modulation. This method has the advantages of low quantization noise, large measuring range, and high linearity [26].

For post-processing techniques, adaptive filtering, Kalman filtering, wavelet transform, artificial neural network, and support vector machine can be used for noise reduction [27,28,29,30]. For instance, Ning Liu et al. proposed a nonlinear suppression method based on cubic Kalman filter-phase space reconstruction (CKF-PSR), which is used to compensate random noise in real time [31]. However, there are difficulties in hardware implementation. An autoregressive Kalman-filter-based model for gyroscope long-term error (LTE) was proposed by Javad Abbasi et al., reducing the zero-bias instability by 50% and the measurement error by 4%~70% [32]. Moreover, the effect of ambient temperature on the performances such as bias instability and scale factor should not be neglected [33,34,35]. In general, high vacuum encapsulation can reduce the air damping and improve the quality factor and mechanical sensitivity. This results in a noise reduction in the detection circuits and control system [36]. In addition, drive closed-loop control techniques such as active gain control (AGC) and phase-locked loop control (PLL) are used to enhance full-temperature control capability [37].

In summary, the weak signals detected by the readout circuits of gyroscopes are inevitably influenced by ambient temperature, mechanical structure, and circuit noises [38]. Noises significantly influence the gyroscope’s mechanical sensitivity, angle random walk, and bias instability [39,40]. In our previous work, an electrical coupling suppression and closed-loop control method was proposed to improve the performance, and the bias instability reached 6.3 deg/h without noise analysis and optimization [41]. However, it can be further advanced by noise analysis and optimization. Therefore, it is essential to analyze the noise contributors in detail and suppress noises from both physical and mathematical perspectives. In [41], the detection and control system was based on both analog and digital circuits, and the analog circuits mainly included the front-end readout circuit, inverting amplifiers, low-pass filters, and high-pass filters, while the digital circuits mainly included the analog to digital converters (ADCs), digital to analog converters (DACs), and field-programmable gate array (FPGA). Given that noises mainly result from the analog circuits, in this paper, the noise analysis and optimization of the key analog circuits will be conducted. Compared with the previous work, this work provides a detailed derivation and analysis of noise in commonly used analog circuits, and integrates methods such as chip selection, circuit structural optimization, and parameter optimization to suppress noise, thus effectively improving the key performance of MEMS gyroscopes.

## 2. Theoretical Analyses

The analog circuits of the drive mode are similar to those of the sense mode; thus, in this work, the analog circuits of the drive mode are taken as the example for analysis.

### 2.1. Noise Analysis of the Readout Circuit

**Figure 1:**
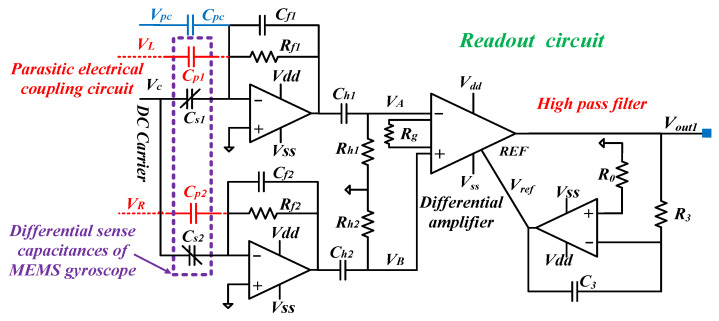
Schematic of the front-end readout circuit with feed-forward coupling compensation control.

In [41], the front-end readout circuit with feed-forward coupling compensation control is illustrated in Figure 1. *V_L_* and *V_R_* are differential drive signals, and *C_s_*_1_ and *C_s_*_2_ are the differential detection capacitor. Their equations can be depicted as (1).

(1) $$\left\{\begin{array}{l} V_{R}=V_{dc}+V_{ac}\sin(\omega_{d}t)\\ V_{L}=V_{dc}-V_{ac}\sin(\omega_{d}t)\\ C_{s1}=\frac{N_{s}\varepsilon hl}{d_{0}}-\frac{N_{s}\varepsilon hA_{d}}{d_{0}}\cos(\omega_{d}t)\\ C_{s2}=\frac{N_{s}\varepsilon hl}{d_{0}}+\frac{N_{s}\varepsilon hA_{d}}{d_{0}}\cos(\omega_{d}t) \end{array}\right.$$

where *ω_d_* is the resonant frequency of the drive mode, and *t* is the time. *N_s_* is the number of detection capacitors, and *ε* is the dielectric constant. *h* is the thickness, *l* is the initial length, and *d*_0_ is the spacing of the combs. *A_d_* is the amplitude of the drive displacement. When the quality factor *Q_d_* is very large, *A_d_* is approximately equal to *F*_0_*Q_d_*/*m_d_ω_d_*^2^. *m_d_* is the drive mass and *F*_0_ is the amplitude of the drive force. *V_ac_* is the amplitude of the drive alternating current (AC) signal, and *V_dc_* is the amplitude of the drive direct current (DC) signal.

*C_p_*_1_ and *C_p_*_2_ are the parasitic capacitors between the drive and sense combs, while *C_pc_* is the coupling compensation capacitor. *V_pc_* is the coupling compensation AC voltage, which is equal to *K_pc_V_ac_*sin(*ω_d_t*). *C_f_*_1_ and *C_f_*_2_ are the feedback capacitors, while *R_f_*_1_ and *R_f_*_2_ are the feedback resistors of the two transimpedance amplifiers. Capacitors *C_h_*_1_ and *C_h_*_2_, and resistors *R_h_*_1_ and *R_h_*_2_ are applied to construct a first-order passive high-pass filter (HPF) to filter the low-frequency signal. *R_g_* is the resistor of the instrumentation amplifier to set a gain of *k_g_*. For avoiding the output saturation of the instrumentation amplifier, the HPF composed of *C*_3_, *R*_3,_ and *R*_0_ is used to eliminate the DC signal.

Assuming that *C_f_*_1_ = *C_f_*_2_ = *C_f_*, *R_f_*_1_ = *R_f_*_2_ = *R_f_*, *C_p_*_1_ = *C_p_*_2_ = *C_p_*, *C_h_*_1_ = *C_h_*_2_ = *C_h_*, *R_h_*_1_ = *R_h_*_2_ = *R_h_*, and 1 << *ω_d_C_h_R_h_*, the output voltage *V_A_*, *V_B,_* and *V_A_* − *V_B_* can be calculated as (2).

(2) $$\left\{\begin{array}{l} V_{A}(s)=-\frac{sR_{f1}(C_{s1}V_{C}+C_{p1}V_{L}+C_{pc}V_{pc})}{1+sR_{f1}C_{f1}}\times \frac{sR_{h1}C_{h1}}{1+sR_{h1}C_{h1}}\\ V_{B}(s)=-\frac{sR_{f2}(C_{s2}V_{C}+C_{p2}V_{R})}{1+sR_{f2}C_{f2}}\times \frac{sR_{h2}C_{h2}}{1+sR_{h2}C_{h2}}\\ V_{B}(s)-V_{A}(s)\approx \frac{sR_{f}[(C_{s1}-C_{s2})V_{C}+C_{p}(V_{L}-V_{R})+C_{pc}V_{pc}]}{1+sR_{f}C_{f}}\\ \;\;\approx \frac{sR_{f}[(K_{pc}C_{pc}-2C_{p})V_{ac}\sin(\omega_{d}t)-\frac{2V_{C}N_{s}\varepsilon hA_{d}}{d_{0}}\cos(\omega_{d}t)]}{1+sR_{f}C_{f}} \end{array}\right.$$

where complex frequency *s* is equal to *jω*, and *ω* stands for the angular frequency. *V_C_* is the DC carrier. Given that the front-end readout circuit adopts the velocity detection scheme, 1 >> *ω_d_C_f_R_f_*. In addition, due to the HPF added to the instrumentation amplifier, 1 << *ω_d_C*_3_*R*_3_. The electrical coupling signals induced by *C_p_*_1_ and *C_p_*_2_ can be effectively suppressed by compensation capacitor *C_pc_*, as long as *K_pc_C_pc_* = 2*C_p_*, as shown in (2). Thus, the output voltage *V_out_*_1_(*s*) can be derived as (3), and the time-domain signal *V_out_*_1_ and its root mean square (RMS) value *V_RMS_*_1_ can be obtained as (4).

(3) $$V_{out1}(s)=k_{g}(V_{B}-V_{A})\times \frac{sR_{3}C_{3}}{1+sR_{3}C_{3}}\approx -\frac{2sk_{g}R_{f}V_{C}N_{s}\varepsilon hA_{d}}{d_{0}}\cos(\omega_{d}t)$$

(4) $$\left\{\begin{array}{l} V_{out1}=\frac{2k_{g}R_{f}V_{C}N_{s}\varepsilon hF_{0}Q_{d}}{d_{0}m_{d}\omega_{d}}\sin(\omega_{d}t)\\ V_{RMS1}=\frac{\sqrt{2}k_{g}R_{f}V_{C}N_{s}\varepsilon hF_{0}Q_{d}}{d_{0}m_{d}\omega_{d}} \end{array}\right.$$

**Figure 2:**
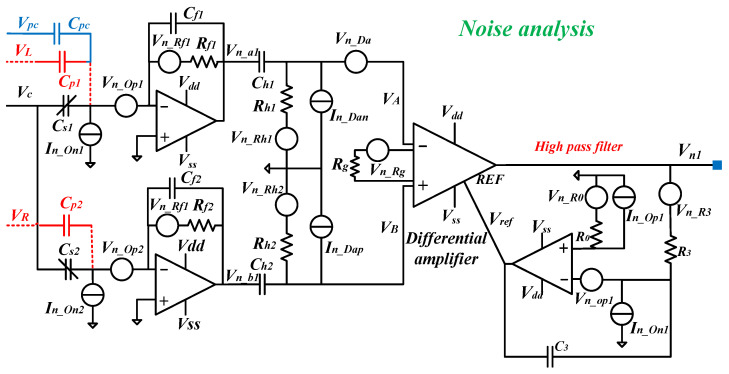
Noise model for a front-end readout circuit with feed-forward coupling compensation control.

The noise analysis schematic of the front-end readout circuit is shown in Figure 2, where *V_n_Op_*_1_ and *V_n_Op_*_2_ are the input noise voltages at the negative end of the two operational amplifiers, *I_n_Op_*_1_ is the input noise current at the positive end of the operational amplifier, *I_n_On_*_1_ and *I_n_On_*_2_ are the input noise currents at the negative end of the two operational amplifiers, *V_n_Da_* is the input noise voltage of the instrumentation amplifier, *I_n_Dap_* and *I_n_Dan_* are the input noise currents at the positive and negative ends of the instrumentation amplifier, and *V_n_Rg_*, *V_n_R_*_3_, *V_n_R_*_0_, *V_n_Rf_*_1_, *V_n_Rf_*_2_, *V_n_Rh_*_1_, and *V_n_Rh_*_2_ are the noise voltages of resistors *R_g_*, *R*_3_, *R*_0_, *R_f_*_1_, *R_f_*_2_, *R_h_*_1_, and *R_h_*_2_, respectively. Based on the definition of Johnson noise, the PSDs of their noise are derived as (5). Thus, the PSDs of the output noise voltage of the two transimpedance amplifiers can be derived as (6). Since the noises of the two transimpedance amplifiers are uncorrelated, the PSD of the output noise voltage of the instrument amplifier can be derived as (7). Assuming that *I_n_Dap_* = *I_n_Dan_* = *I_n_Da_*, *V_n_Op_*_1_ = *V_n_Op_*_2_ = *V_n_Op_*, *I_n_On_*_1_ = *I_n_On_*_2_ = *I_n_On_*, *I_n_Op_*_1_ = *I_n_Op_*_2_ = *I_n_Op_*, and considering the noise at the resonant frequency *ω_d_*, then (6) and (7) can be simplified to (8) and (9), respectively.

(5) $$\left\{\begin{array}{l} \frac{V_{n\_Rg}}{\sqrt{\Delta f}}=\sqrt{4k_{b}TR_{g}},\frac{V_{n\_R3}}{\sqrt{\Delta f}}=\sqrt{4k_{b}TR_{3}},\frac{V_{n\_R0}}{\sqrt{\Delta f}}=\sqrt{4k_{b}TR_{0}},\\ \frac{V_{n\_Rf1}}{\sqrt{\Delta f}}=\sqrt{4k_{b}TR_{f1}},\frac{V_{n\_Rf2}}{\sqrt{\Delta f}}=\sqrt{4k_{b}TR_{f2}},\\ \frac{V_{n\_Rh1}}{\sqrt{\Delta f}}=\sqrt{4k_{b}TR_{h1}},\frac{V_{n\_Rh2}}{\sqrt{\Delta f}}=\sqrt{4k_{b}TR_{h2}} \end{array}\right.$$

(6) $$\left\{\begin{array}{l} \frac{V_{n\_a1}^{2}}{\Delta f}=\frac{4k_{b}TR_{f1}}{1+\omega^{2}R_{f1}^{2}C_{f1}^{2}}+\frac{I_{n\_On1}^{2}R_{f1}^{2}}{1+\omega^{2}R_{f1}^{2}C_{f1}^{2}}\\ \;\;+\frac{V_{n\_Op1}^{2}[1+\omega^{2}R_{f1}^{2}{\left(C_{f1}+C_{s1}+C_{p1}+C_{pc}\right)}^{2}]}{1+\omega^{2}R_{f1}^{2}C_{f1}^{2}}\\ \frac{V_{n\_b1}^{2}}{\Delta f}=\frac{4k_{b}TR_{f2}}{1+\omega^{2}R_{f2}^{2}C_{f2}^{2}}+\frac{I_{n\_On2}^{2}R_{f2}^{2}}{1+\omega^{2}R_{f2}^{2}C_{f2}^{2}}\\ \;\;+\frac{V_{n\_Op2}^{2}[1+\omega^{2}R_{f2}^{2}{\left(C_{f2}+C_{s2}+C_{p2}\right)}^{2}]}{1+\omega^{2}R_{f2}^{2}C_{f2}^{2}} \end{array}\right.$$

(7) $$\left\{\begin{array}{l} \frac{V_{n1}^{2}}{\Delta f}=\frac{V_{n10}^{2}}{\Delta f}\frac{\omega^{2}R_{3}^{2}C_{3}^{2}}{1+\omega^{2}R_{3}^{2}C_{3}^{2}}+\frac{1+\omega^{2}R_{3}^{2}C_{3}^{2}}{\omega^{2}R_{3}^{2}C_{3}^{2}}(4k_{b}TR_{0}+I_{n\_Op1}^{2}R_{0}^{2}+V_{n\_Op1}^{2})\\ \;\;\;+\frac{4k_{b}TR_{3}}{\omega^{2}R_{3}^{2}C_{3}^{2}}+\frac{I_{n\_On1}^{2}}{\omega^{2}C_{3}^{2}}\\ \frac{V_{n10}^{2}}{\Delta f}=k_{g}^{2}[\frac{V_{n\_a1}^{2}}{\Delta f}\frac{\omega^{2}R_{h1}^{2}C_{h1}^{2}}{1+\omega^{2}R_{h1}^{2}C_{h1}^{2}}+\frac{V_{n\_b1}^{2}}{\Delta f}\frac{\omega^{2}R_{h2}^{2}C_{h2}^{2}}{1+\omega^{2}R_{h2}^{2}C_{h2}^{2}}+\frac{4k_{b}TR_{h1}}{1+\omega^{2}R_{h1}^{2}C_{h1}^{2}}\\ \;+\frac{4k_{b}TR_{h2}}{1+\omega^{2}R_{h2}^{2}C_{h2}^{2}}+\frac{I_{n\_Dan}^{2}R_{h1}^{2}}{1+\omega^{2}R_{h1}^{2}C_{h1}^{2}}+\frac{I_{n\_Dap}^{2}R_{h2}^{2}}{1+\omega^{2}R_{h2}^{2}C_{h2}^{2}}+V_{n\_Da}^{2}+4k_{b}TR_{g}] \end{array}\right.$$

(8) $$\left\{\begin{array}{l} \frac{V_{n\_a1}^{2}}{\Delta f}\approx 4k_{b}TR_{f1}+I_{n\_On1}^{2}R_{f1}^{2}+V_{n\_Op1}^{2}\\ \;\;\;=4k_{b}TR_{f}+I_{n\_On}^{2}R_{f}^{2}+V_{n\_Op}^{2}\\ \frac{V_{n\_b1}^{2}}{\Delta f}\approx 4k_{b}TR_{f2}+I_{n\_On2}^{2}R_{f2}^{2}+V_{n\_Op2}^{2}\\ \;\;\;=4k_{b}TR_{f}+I_{n\_On}^{2}R_{f}^{2}+V_{n\_Op}^{2} \end{array}\right.$$

(9) $$\left\{\begin{array}{l} \frac{V_{n1}^{2}}{\Delta f}=\frac{V_{n10}^{2}}{\Delta f}+(4k_{b}TR_{0}+I_{n\_Op}^{2}R_{0}^{2}+V_{n\_Op}^{2})+\frac{4k_{b}TR_{3}}{\omega_{d}^{2}R_{3}^{2}C_{3}^{2}}+\frac{I_{n\_On}^{2}}{\omega_{d}^{2}C_{3}^{2}}\\ \frac{V_{n10}^{2}}{\Delta f}=k_{g}^{2}[2\times (4k_{b}TR_{f}+I_{n\_On}^{2}R_{f}^{2}+V_{n\_Op}^{2}+\frac{4k_{b}T}{\omega_{d}^{2}R_{h}C_{h}^{2}}+\frac{I_{n\_Da}^{2}}{\omega_{d}^{2}C_{h}^{2}})\\ \;\;\;+V_{n\_Da}^{2}+4k_{b}TR_{g}] \end{array}\right.$$

Here, *R*_0_ is a matching resistor. Due to the inevitable input offset current in the operational amplifier, it is necessary to make the DC channel resistance at both ends of the operational amplifier equal, namely, *R*_0_ = *R*_3_, so as to balance the input bias current. Fortunately, the operational amplifier is applied to construct an HPF; thus, the mismatch caused by the input bias current can be filtered by the subsequent HPF and the signal demodulation. Therefore, *R*_0_ should be set to 0 to minimize the output noise shown in (9). Thus, (9) can be simplified to (10).

(10) $$\frac{V_{n1}}{\sqrt{\Delta f}}=\sqrt{\begin{array}{l} k_{g}^{2}[2\times (4k_{b}TR_{f}+I_{n\_On}^{2}R_{f}^{2}+V_{n\_Op}^{2}+\frac{4k_{b}T}{\omega_{d}^{2}R_{h}C_{h}^{2}}+\frac{I_{n\_Da}^{2}}{\omega_{d}^{2}C_{h}^{2}})\\ \;+V_{n\_Da}^{2}+4k_{b}TR_{g}]+V_{n\_Op}^{2}+\frac{4k_{b}TR_{3}}{\omega_{d}^{2}R_{3}^{2}C_{3}^{2}}+\frac{I_{n\_On}^{2}}{\omega_{d}^{2}C_{3}^{2}} \end{array}}$$

To further suppress the circuit noise, it is necessary to select precision operational amplifiers and instrumentation amplifiers with high bandwidth, high slew rate, and low noise, so as to minimize the PSDs of input noise voltage and current. In [41], OP2177 and AD8221 are selected as the operational amplifiers and instrumentation amplifier, respectively. They are both powered by Analog Devices, Inc., Norwood, MA, USA. The bandwidth of OP2177 is 1.3 MHz, the slew rate is 0.7 V/μs, the input voltage noise density is 7.9 nV/√Hz, and the input current noise density is 0.2 pA/√Hz. The bandwidth gain product of AD8221 is 0.825 MHz, the slew rate is 2 V/μs, the input voltage noise density is 8 nV/√Hz, and the input current noise density is 6 pA/√Hz. However, they are not the best chips, and they can be replaced by AD8676 and AD8421, respectively. These two chip are also both powered by Analog Devices, Inc., USA. The bandwidth of AD8676 is 10 MHz, the slew rate is 2.5 V/μs, the input voltage noise density is 2.8 nV/√Hz, and the input current noise density is about 0.2 pA/√Hz. The bandwidth gain product of AD8221 is 10 MHz, the slew rate is 35 V/μs, the input voltage noise density is 3 nV/√Hz, and the input current noise density is 0.2 pA/√Hz. It is obvious that chip selection is very important.

In addition, for suppressing the noise, *ω_d_*, *C_h_*, and *R_h_* should be increased appropriately, while *k_g_*, *R_g_*, and *R_f_* should be decreased. Considering that *R_g_* and *k_g_* are inversely proportional, they cannot be increased or decreased simultaneously. If *k_g_* and *R_f_* are reduced, the gain of the signal will be also decreased. Here, AD8676 and AD8421 are selected, that is, *I_n_On_* = 0.2 pA/√Hz, *V_n_Op_* = 2.8 nV/√Hz, *I_n_Da_* = 0.2 pA/√Hz, and *V_n_Da_* = 3 nV/√Hz. In addition, *k_b_* = 1.38 × 10^−23^ J/K, *T* = 300 K, *ω_d_* = 2π × 8410.6 rad/s, *R_f_* = 1 MΩ, *C_f_* = 1 pF, *R_h_* = 200 kΩ, *C_h_* = 0.01 μF, *R_g_* = 100 Ω. Based on (10) and data calculation, it is clear that the first two terms are the main noises, which are much larger than the other terms. Thus, (10) can be simplified to (11).

(11) $$\frac{V_{n1}}{\sqrt{\Delta f}}\approx k_{g}\sqrt{2\times (4k_{b}TR_{f}+I_{n\_On}^{2}R_{f}^{2})}$$

Therefore, based on (4) and (11), the signal-to-noise ratio *SNR*_1_ of the front-end readout circuit can be obtained as (12).

(12) $$SNR_{1}=\frac{V_{RMS1}}{\frac{V_{n1}}{\sqrt{\Delta f}}}\approx \frac{V_{C}N_{s}\varepsilon hF_{0}Q_{d}}{d_{0}m_{d}\omega_{d}\sqrt{4k_{b}T/R_{f}+I_{n\_On}^{2}}}$$

It is obvious that to improve the SNR of the front-end readout circuit, *V_C_*, *h*, *F*_0_, *Q_d_*, and *R_f_* should be appropriately increased, while *d*_0_, *m_d_*, *ω_d_*, and *I_n_On_* should be reduced. Therefore, in addition to optimizing the structural design and selecting the precise operational amplifier, it is also necessary to set the feedback resistance *R_f_* reasonably. Here, circuit improvement can be achieved with the T-type resistor networks, which can further suppress the noise and advance the SNR.

### 2.2. An Improved Readout Circuit Based on the T-Resistor Networks

**Figure 3:**
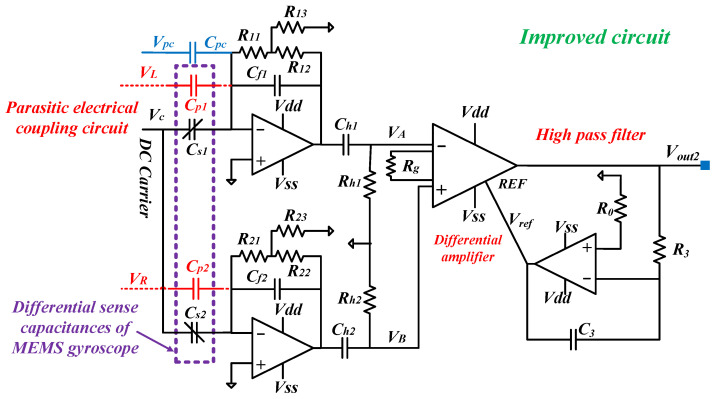
Schematic of an improved front-end readout circuit based on T-resistor networks.

The readout circuit is improved by the T-resistor networks, as shown in Figure 3. Assuming that *R*_11_ = *R*_21_, *R*_12_ = *R*_22_, and *R*_13_ = *R*_23_, then the output voltage *V_out_*_2_ and its root mean square (RMS) value *V_RMS_*_2_ can be similarly calculated as (13).

(13) $$\left\{\begin{array}{l} V_{out2}=\frac{2k_{g}R_{ff}V_{C}N_{s}\varepsilon hF_{0}Q_{d}}{d_{0}m_{d}\omega_{d}}\sin(\omega_{d}t)\\ V_{RMS2}=\frac{\sqrt{2}k_{g}R_{ff}V_{C}N_{s}\varepsilon hF_{0}Q_{d}}{d_{0}m_{d}\omega_{d}} \end{array}\right.$$

where *R_ff_* = *R*_11_ + *R*_12_ + *R*_11_*R*_12_/*R*_13_. Given that the front-end readout circuit adopts the velocity detection scheme, 1 >> *ω_d_C_f_R_ff_*. Assuming that *R*_11_ = 100 kΩ, *R*_12_ = 1 kΩ, *R*_13_ = 110Ω, then *R_ff_* ≈ 1 MΩ = *R_f_*. It means that the improved circuit and the original circuit have the same transimpedance.

**Figure 4:**
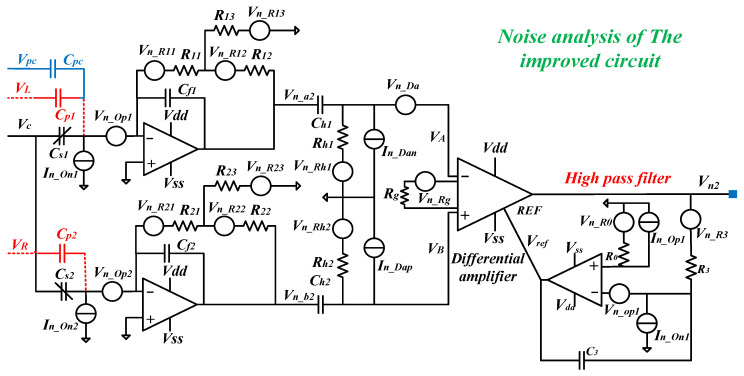
Noise analysis for an improved front-end readout circuit based on T-resistor networks.

The noise analysis schematic of the improved front-end readout circuit is shown in Figure 4. The noise analysis and derivation are similar to those above. Thus, the PSD of the output noise voltage of the upper transimpedance amplifier can be derived as (14).

(14) $$\begin{array}{l} \frac{V_{na2}^{2}}{\Delta f}=\frac{I_{n\_On1}^{2}R_{ff}^{2}}{1+\omega^{2}R_{ff}^{2}C_{f1}^{2}}+\frac{4k_{b}TR_{11}}{{\left(1+R_{11}/R_{13}\right)}^{2}+\omega^{2}R_{ff}^{2}C_{f1}^{2}}+\frac{4k_{b}TR_{12}}{1+\omega^{2}R_{12}^{2}C_{f1}^{2}}\\ \;\;+\frac{4k_{b}TR_{13}}{{\left(1+R_{13}/R_{11}\right)}^{2}+\omega^{2}{\left(R_{13}+R_{12}+R_{13}R_{12}/R_{11}\right)}^{2}C_{f1}^{2}}\\ \;\;+\frac{V_{n\_Op}^{2}[{\left(1+\frac{R_{12}}{R_{13}}\right)}^{2}+\omega^{2}R_{ff}^{2}{\left(C_{f1}+C_{s1}+C_{p1}+C_{pc}\right)}^{2}]}{1+\omega^{2}R_{ff}^{2}C_{f1}^{2}} \end{array}$$

Similarly, considering the noise at the resonant frequency *ω_d_*, then the (14) can be simplified as (15).

(15) $$\frac{V_{na2}^{2}}{\Delta f}\approx 4k_{b}T(R_{13}^{2}/R_{11}+R_{13}+R_{12})+I_{n\_On}^{2}R_{ff}^{2}+V_{n\_Op}^{2}R_{12}^{2}/R_{13}^{2}$$

Likewise, the PSD of the output noise voltage of the lower transimpedance amplifier can be derived as (16).

(16) $$\frac{V_{nb2}^{2}}{\Delta f}\approx 4k_{b}T(R_{13}^{2}/R_{11}+R_{13}+R_{12})+I_{n\_On}^{2}R_{ff}^{2}+V_{n\_Op}^{2}R_{12}^{2}/R_{13}^{2}$$

Thus, the PSD of the output noise voltage of the instrument amplifier can be derived as (17).

(17) $$\left\{\begin{array}{l} \frac{V_{n2}^{2}}{\Delta f}=\frac{V_{n20}^{2}}{\Delta f}+(4k_{b}TR_{0}+I_{n\_Op}^{2}R_{0}^{2}+V_{n\_Op}^{2})+\frac{4k_{b}TR_{3}}{\omega_{d}^{2}R_{3}^{2}C_{3}^{2}}+\frac{I_{n\_On}^{2}}{\omega_{d}^{2}C_{3}^{2}}\\ \frac{V_{n20}^{2}}{\Delta f}=k_{g}^{2}\{2\times [4k_{b}T(R_{13}^{2}/R_{11}+R_{13}+R_{12})+I_{n\_On}^{2}R_{ff}^{2}\\ \;\;\;+V_{n\_Op}^{2}R_{12}^{2}/R_{13}^{2}+\frac{4k_{b}T}{\omega_{d}^{2}R_{h}C_{h}^{2}}+\frac{I_{n\_Da}^{2}}{\omega_{d}^{2}C_{h}^{2}}]+V_{n\_Da}^{2}+4k_{b}TR_{g}\} \end{array}\right.$$

Likewise, *R*_0_ should be set to 0 to minimize the output noise shown in (9). Thus (17) can be simplified to (18).

(18) $$\frac{V_{n2}}{\sqrt{\Delta f}}=\sqrt{\begin{array}{l} k_{g}^{2}\{2\times [4k_{b}T(R_{13}^{2}/R_{11}+R_{13}+R_{12})+I_{n\_On}^{2}R_{ff}^{2}\\ \;+V_{n\_Op}^{2}R_{12}^{2}/R_{13}^{2}+\frac{4k_{b}T}{\omega_{d}^{2}R_{h}C_{h}^{2}}+\frac{I_{n\_Da}^{2}}{\omega_{d}^{2}C_{h}^{2}}]+V_{n\_Da}^{2}\\ \;+4k_{b}TR_{g}\}+V_{n\_Op}^{2}+\frac{4k_{b}TR_{3}}{\omega_{d}^{2}R_{3}^{2}C_{3}^{2}}+\frac{I_{n\_On}^{2}}{\omega_{d}^{2}C_{3}^{2}} \end{array}}$$

After calculation, it is clear that the first two terms are the main noises, which are much larger than the other terms. Thus, (18) can be simplified to (19).

(19) $$\frac{V_{n2}}{\sqrt{\Delta f}}\approx k_{g}\sqrt{2\times [4k_{b}T(R_{13}^{2}/R_{11}+R_{13}+R_{12})+I_{n\_On}^{2}R_{ff}^{2}]}$$

Therefore, based on (13) and (19), the signal-to-noise ratio *SNR*_2_ of the front-end readout circuit can be obtained as (20).

(20) $$SNR_{2}=\frac{V_{RMS2}}{\frac{V_{n2}}{\sqrt{\Delta f}}}\approx \frac{V_{C}N_{s}\varepsilon hF_{0}Q_{d}/(d_{0}m_{d}\omega_{d})}{\sqrt{4k_{b}T(R_{13}^{2}/R_{11}+R_{13}+R_{12})/R_{ff}^{2}+I_{n\_On}^{2}}}$$

From (4) and (13), the gain of the improved circuit with T-resistor networks remains unchanged. However, from (12) and (20), the SNR of the improved circuit has been advanced because $(R_{13}^{2}/R_{11}+R_{13}+R_{12})/R_{ff}^{2}\approx R_{12}/R_{ff}^{2}<1/R_{ff}$. In addition, the T-resistor network can achieve a high feedback resistance using only three resistors with small resistances, and a single resistor with a very high resistance is hard to achieve. Thus, the noise analysis above figures out that the improved circuit with T-resistor networks are effective to reduce the circuit noise.

### 2.3. Noise Analysis of the Other Critical Circuits

Given the importance of the inverting amplifier, first-order high-pass filter, and first-order low-pass filter (LPF) in the front-end readout circuit, it is imperative to give careful consideration to the noises induced by these circuits.

**Figure 5:**
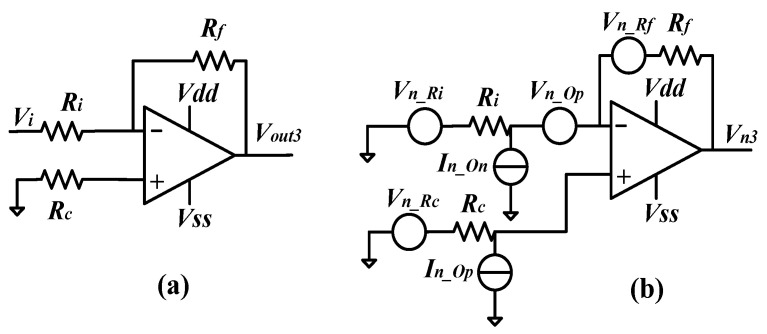
(**a**) Schematic of the inverting amplifier; (**b**) noise analysis for the inverting amplifier.

The circuit schematic and noise analysis schematic of the inverting amplifier are illustrated in Figure 5a,b, respectively. Based on the circuits, the gain and output noise can be derived as (21) and (22).

(21) $$\frac{V_{out3}}{V_{i}}=\frac{-R_{f}}{R_{i}}$$

(22) $$\begin{array}{l} \frac{V_{n3}^{2}}{\Delta f}={\left(1+R_{f}/R_{i}\right)}^{2}(4k_{b}TR_{c}+I_{n\_Op}^{2}R_{c}^{2}+V_{n\_Op}^{2})\\ \;\;\;+4k_{b}TR_{f}+4k_{b}TR_{i}{\left(R_{f}/R_{i}\right)}^{2}+I_{n\_On}^{2}R_{f}^{2} \end{array}$$

where *V_out_*_3_ and *V_n_*_3_ are the output voltage and noise voltage of the inverting amplifier, respectively. *R_c_* is a matching resistor. Due to the inevitable input offset current in the operational amplifier, it is necessary to make the DC channel resistance at both ends of the operational amplifier equal, namely, *R_c_* = *R_i_*//*R_f_*, so as to balance the input bias current. Fortunately, the mismatch caused by the input bias current can be filtered by the subsequent HPF and the signal demodulation. Therefore, *R_c_* should be set to 0 to minimize the output noise. Thus, the SNR can be obtained as (23).

(23) $$SNR_{3}=\frac{V_{out3}}{\frac{V_{n3}}{\sqrt{\Delta f}}}=\frac{V_{i}}{\sqrt{4k_{b}T(R_{i}+R_{i}^{2}/R_{f})+{\left(1+R_{i}/R_{f}\right)}^{2}V_{n\_Op}^{2}+R_{i}^{2}I_{n\_On}^{2}}}$$

**Figure 6:**
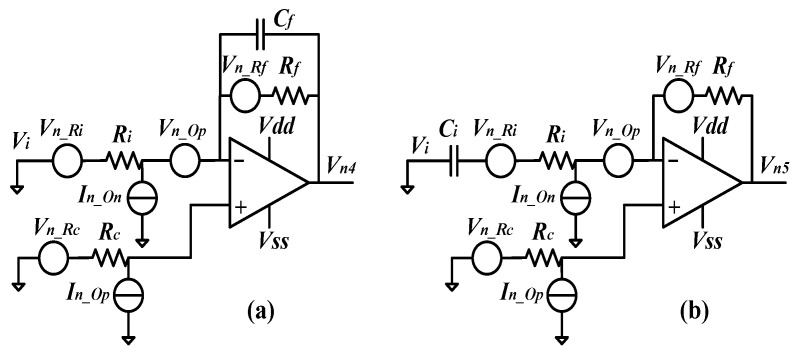
(**a**) Noise analysis for the first-order low-pass filter; (**b**) noise analysis for the first-order high-pass filter.

Similarly, the noise analysis schematics of the first-order LPF and the first-order HPF are shown in Figure 6a,b. For the LPF, the transfer function and the PSD of the output noise voltage can be obtained as (24) and (25), respectively. Likewise, to suppress the noise, *R_c_* should be set to 0. If the noise within the passband is considered, thus 1 >> *ωC_f_R_f_*, and the simplified SNR can be obtained as (26).

(24) $$\frac{V_{out4}}{V_{i}}=\frac{-R_{f}/R_{i}}{1+sR_{f}C_{f}}$$

(25) $$\begin{array}{l} \frac{V_{n4}^{2}}{\Delta f}=\frac{4k_{b}TR_{f}}{1+\omega^{2}R_{f}^{2}C_{f}^{2}}+\frac{4k_{b}TR_{i}{\left(R_{f}/R_{i}\right)}^{2}}{1+\omega^{2}R_{f}^{2}C_{f}^{2}}+\frac{I_{n\_On}^{2}R_{f}^{2}}{1+\omega^{2}R_{f}^{2}C_{f}^{2}}\\ \;\;\;+\frac{{\left(1+R_{f}/R_{i}\right)}^{2}+\omega^{2}R_{f}^{2}C_{f}^{2}}{1+\omega^{2}R_{f}^{2}C_{f}^{2}}(4k_{b}TR_{c}+I_{n\_Op}^{2}R_{c}^{2}+V_{n\_Op}^{2}) \end{array}$$

(26) $$SNR_{4}=\frac{V_{out4}}{\frac{V_{n4}}{\sqrt{\Delta f}}}\approx \frac{V_{i}}{\sqrt{4k_{b}T(R_{i}+R_{i}^{2}/R_{f})+{\left(1+R_{i}/R_{f}\right)}^{2}V_{n\_Op}^{2}+R_{i}^{2}I_{n\_On}^{2}}}$$

where *V_out_*_4_ and *V_n_*_4_ are the output voltage and noise voltage of the first-order LPF, respectively. For the HPF, the transfer function and the PSD of the output noise voltage can be obtained as (27) and (28), respectively. Likewise, to suppress the noise, *R_c_* should be set to 0. If the noise within the passband is considered, 1 >> *ωC_f_R_f_*, and the simplified SNR can be obtained as (29).

(27) $$\frac{V_{out5}}{V_{i}}=\frac{-sR_{f}C_{i}}{1+sR_{i}C_{i}}$$

(28) $$\begin{array}{l} \frac{V_{n5}^{2}}{\Delta f}=4k_{b}TR_{f}+4k_{b}TR_{i}{\left(R_{f}/R_{i}\right)}^{2}+I_{n\_On}^{2}R_{f}^{2}\\ \;\;\;+\frac{1+\omega^{2}{\left(R_{f}+R_{i}\right)}^{2}C_{i}^{2}}{1+\omega^{2}R_{i}^{2}C_{i}^{2}}(4k_{b}TR_{c}+I_{n\_Op}^{2}R_{c}^{2}+V_{n\_Op}^{2}) \end{array}$$

(29) $$SNR_{5}=\frac{V_{out5}}{\frac{V_{n5}}{\sqrt{\Delta f}}}\approx \frac{V_{i}}{\sqrt{4k_{b}T(R_{i}+R_{i}^{2}/R_{f})+{\left(1+R_{i}/R_{f}\right)}^{2}V_{n\_Op}^{2}+R_{i}^{2}I_{n\_On}^{2}}}$$

where *V_out_*_5_ and *V_n_*_5_ are the output voltage and noise voltage of the first-order HPF, respectively.

It is clear that the expressions of (23), (26) and (29) are the same, and the SNR is closely related to the performances of the operational amplifier and the values of the resistors. Therefore, to suppress the noise, the high-precision operational amplifier with ultra-low *I_n_On_* and *V_n_Op_* should be selected. Additionally, it is important to minimize *R_i_* and *R_i_*/*R_f_*. However, decreasing *R_i_*/*R_f_* leads to a higher circuit gain and power consumption. Therefore, a compromise scheme of parameter setting should be selected.

## 3. Experimental Results

In the previous section, a detailed noise analysis was conducted on the key analog circuits. Here, the inverting amplifier and the gyroscope system are taken as the examples for experimental verification.

**Figure 7:**
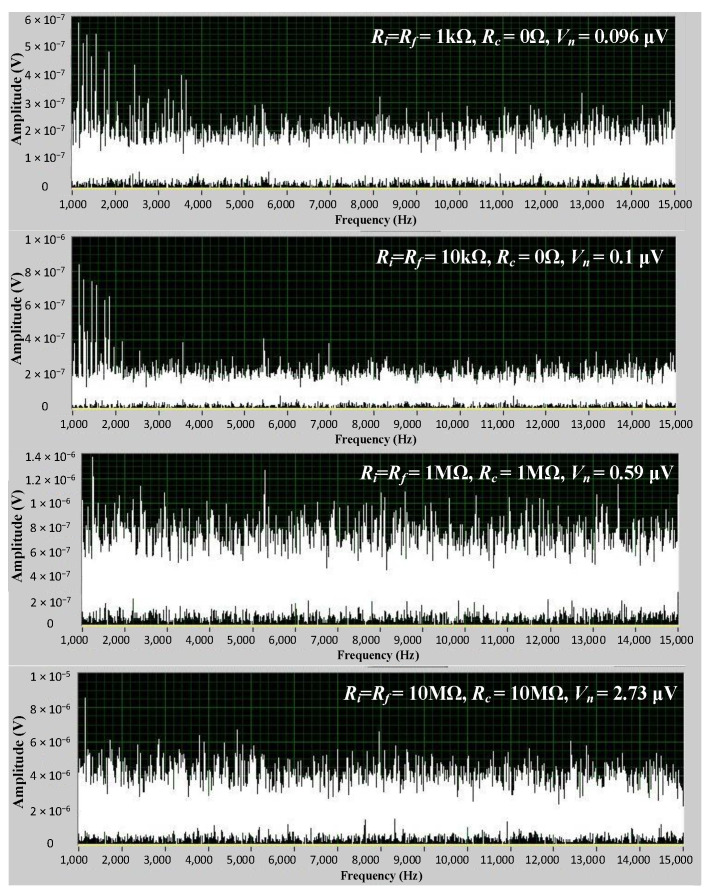
The output noise signal is measured by LabVIEW software when *R_i_*, *R_f_*, and *R_c_* are adjusted.

For the inverting amplifier, to study the influence of different resistances (i.e., *R_i_*, *R_f_*, and *R_c_*) on the output noise or SNR of the inverting amplifier, the output noise signal is measured by LabVIEW2015 software when *R_i_*, *R_f_*, and *R_c_* are adjusted, as shown in Figure 7. The models of the operational amplifier and instrumentation amplifier in the tests are AD8676 and AD8421 from Analog Devices, Inc., respectively. In order to facilitate the analysis, *R_i_* is set to equal *R_f_*, so that the gain is 1. Based on the output noise signal, the RMS of the noise can be calculated. Therefore, for different resistance schemes, the RMS of the output noise can be obtained, as listed in Table 1. In this table, the minimum value is 0.096 μV, while the maximum is 2.73 μV. The gap is very large, which indicates that the different parameter setting schemes have a significant impact on the output noise. When *R_f_* and *R_i_* remain constant, the smaller *R_c_* is, the smaller the output noise is. When *R_i_*/*R_f_* and *R_c_* remain constant, the smaller *R_i_* is, and the smaller the output noise is. These test results accord with those of the theoretical analysis above. For other key analog circuits, the conclusions are also similar, so we will not list them one by one. Next, we focus on the noise optimization verification of the whole gyroscope.

**Figure 8:**
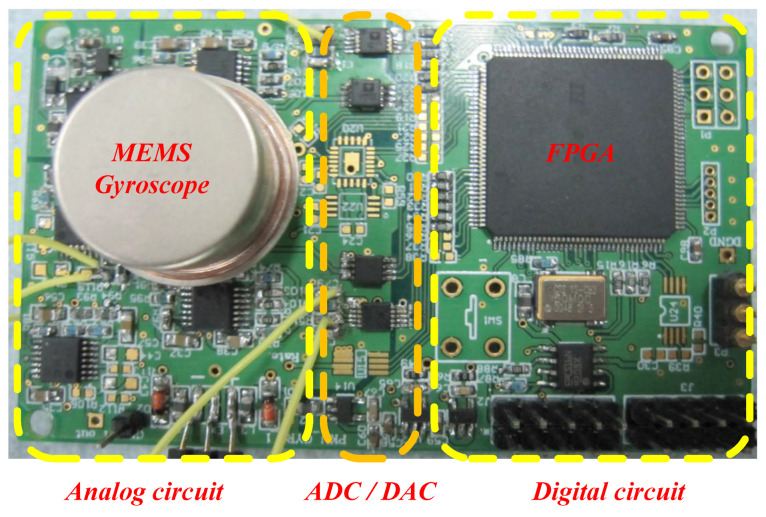
The detection and control system for an MEMS gyroscope.

Subsequently, the output noises of the gyroscope before and after circuit optimization are measured. The chip selection and parameter setting before and after circuit optimization are depicted in Table 2. The measured results are larger than the theoretical results due to the measurement noise and resolution of the measuring instrument. The detection and control system for an MEMS gyroscope is illustrated in Figure 8. It can be used to realize the system with or without circuit optimization. It is mainly composed of an analog circuit, a digital circuit, and an MEMS gyroscope. The experimental sample is an MEMS gyroscope, namely, model 3#621, fabricated by the National Key Laboratory of Science and Technology on Micro/Nano Fabrication at Peking University. The details about the MEMS gyroscope and the system were given in our previous work [41].

**Figure 9:**
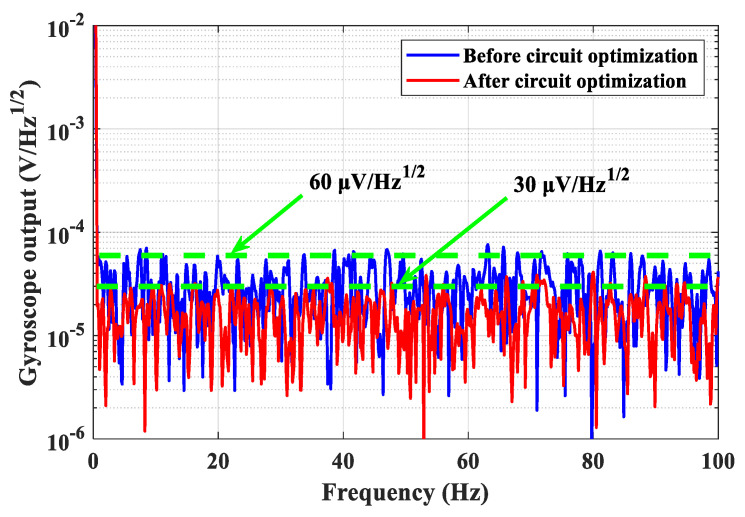
The output noise spectrums of the gyroscope before and after circuit optimization.

**Figure 10:**
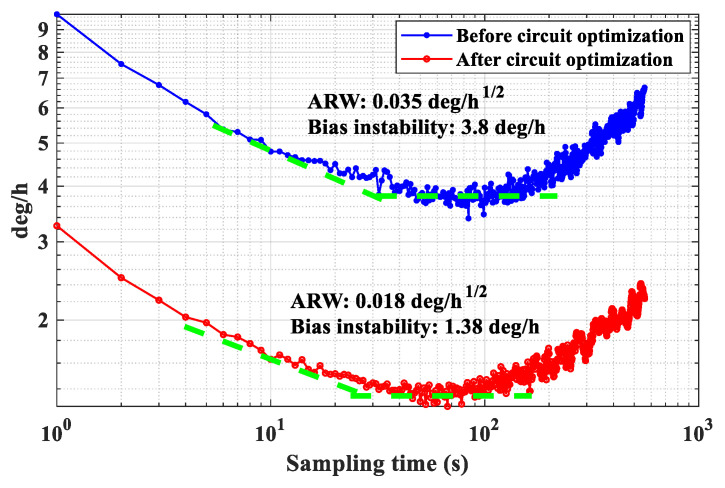
Allan variance curves of the gyroscope before and after circuit optimization.

The output noise spectrums of the gyroscope before and after circuit optimization are illustrated in Figure 9. It shows that before circuit optimization, the maximum output noise is about 60 μV/Hz^1/2^. However, after circuit optimization, it can be significantly reduced to about 30 μV/Hz^1/2^. In addition, Allan variance is adopted to analyze the noise of the gyroscope, as shown in Figure 10. Before circuit optimization, the bias instability and ARW are 3.8 deg/h and 0.035 deg/h^1/2^, respectively. However, after circuit optimization, they are improved to 1.38 deg/h and 0.018 deg/h^1/2^, respectively. Thus, the proposed noise analysis and suppression methods for the detection and control circuits of an MEMS gyroscope are feasible and effective. In addition, it should be noted that due to limitations in amplification or cut-off frequency requirements, the output noise cannot be infinitely reduced and can only be optimized within a limited range.

## 4. Conclusions

In this paper, an optimized front-end readout circuit and noise suppression methods for an MEMS gyroscope were proposed. To suppress the output noise, an improved readout circuit based on the T-resistor networks was proposed, and the corresponding noise equation was derived in detail. In addition, the noise analysis of the key analog circuits, such as the inverting amplifiers, the first-order low-pass filters, and the first-order high-pass filters, was carried out, and the noise suppression strategy with the optimization of the resistances was proposed. Taking the inverting amplifier as an example, the theoretical derivation was verified by measuring and comparing the output noises of different resistance schemes. In addition, the output noises of the gyroscope before and after circuit optimization were measured. Experimental results demonstrated that the output noise with the circuit optimization was reduced from 60 μV/Hz^1/2^ to 30 μV/Hz^1/2^ and the bias instability was reduced from 3.8 deg/h to 1.38 deg/h. In addition, the ARW was significantly improved from 0.035 deg/h^1/2^ to 0.018 deg/h^1/2^, which indicates that the noise analysis and suppression methods were effective. This paper mainly analyzed and improved the noises of the key analog circuits. In the future, a detailed analysis of the noises of the critical digital circuits will be conducted. In addition, the individual contribution of each method to the noise reduction and more optimization strategies will be considered to further improve the performances of the MEMS gyroscope.

## Figures and Tables

**Table 1:** The noise test and theoretical results of different resistance schemes.

Test	*R*_i_ (Ω)	*R*_f_ (Ω)	*R*_c_ (Ω)	Test Result *V*_n_ (μV)
1	1 k	1 k	0	0.096
2	1 k	1 k	10 M	3
3	10 k	10 k	0	0.1
4	10 k	10 k	10 k	0.11
5	100 k	100 k	0	0.13
6	100 k	100 k	100 k	0.19
7	1 M	1 M	0	0.35
8	1 M	1 M	1 M	0.59
9	10 M	10 M	0	1.98
10	10 M	10 M	10 M	2.73

**Table 2:** The chip selection and parameter setting before and after circuit optimization.

Type	Before Circuit Optimization	After Circuit Optimization
Chip selection	OP2177, AD8221	AD8676, AD8421
Transimpedance amplifier	Without T-resistor networks *R*_f_ = 1 MΩ, *C*_f_ = 1 pF	With T-resistor networks *R*_11_ = 100 kΩ, *R*_12_ = 1 kΩ, *R*_13_ = 110 Ω, *C*_f_ = 1 pF
Inverting amplifier	*R*_f_ = 100 kΩ, *R*_i_ = 100 kΩ, *R*_c_ = 100 kΩ	*R*_f_ = 1 kΩ, *R*_i_ = 1 kΩ, *R*_c_ = 0 Ω
LPF	*R*_f_ = 200 kΩ, *R*_i_ = 200 kΩ, *C*_i_ = 1 pF, Rc = 100 kΩ	*R*_f_ = 2 kΩ, *R*_i_ = 2 kΩ, *C*_i_ = 100 pF, *R*_c_ = 0 Ω
HPF	*R*_f_ = 200 kΩ, *R*_i_ = 200 kΩ, *C*_i_ = 0.01 μF, *R*_c_ = 100 kΩ	*R*_f_ = 2 kΩ, *R*_i_ = 2 kΩ, *C*_i_ = 1 μF, *R*_c_ = 0 Ω

## Data Availability

The authors do not have permission to share data.
